# Supplementary material for: Mutations in PCYT2 disrupt etherlipid biosynthesis and cause a complex hereditary spastic paraplegia
Source: Brain. 2019 Oct 22;142(11):3382–97. doi: 10.1093/brain/awz291 (PMC6821184; doi:10.1093/brain/awz291)
Supplement: awz291_Supplementary_Data [file awz291_supplementary_data.zip › awz291-Suppl_data/Supplementary_Data4.zip!/Supplementary information 4 - plasma lipidomics.zip!/Plasma lipidomics/Control vs Patient3b/Statistical plots and data/Random forest.pdf]

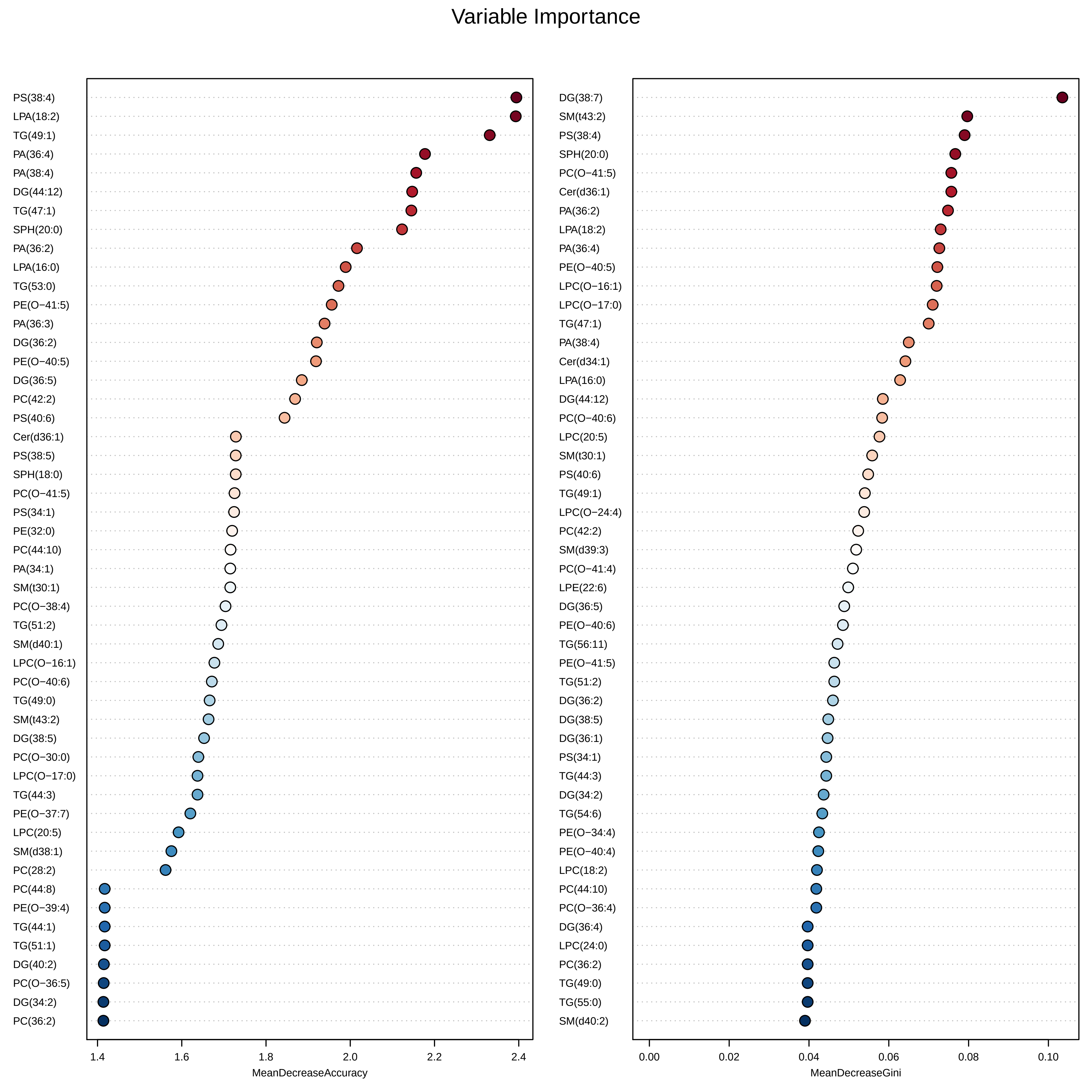

Variable Importance
PS(38:4)
LPA(18:2)
TG(49:1)
PA(36:4)
PA(38:4)
DG(44:12)
TG(47:1)
SPH(20:0)
PA(36:2)
LPA(16:0)
TG(53:0)
PE(O−41:5)
PA(36:3)
DG(36:2)
PE(O−40:5)
DG(36:5)
PC(42:2)
PS(40:6)
Cer(d36:1)
PS(38:5)
SPH(18:0)
PC(O−41:5)
PS(34:1)
PE(32:0)
PC(44:10)
PA(34:1)
SM(t30:1)
PC(O−38:4)
TG(51:2)
SM(d40:1)
LPC(O−16:1)
PC(O−40:6)
TG(49:0)
SM(t43:2)
DG(38:5)
PC(O−30:0)
LPC(O−17:0)
TG(44:3)
PE(O−37:7)
LPC(20:5)
SM(d38:1)
PC(28:2)
PC(44:8)
PE(O−39:4)
TG(44:1)
TG(51:1)
DG(40:2)
PC(O−36:5)
DG(34:2)
PC(36:2)
1.4
1.6
1.8
2.0
2.2
2.4
MeanDecreaseAccuracy
DG(38:7)
SM(t43:2)
PS(38:4)
SPH(20:0)
PC(O−41:5)
Cer(d36:1)
PA(36:2)
LPA(18:2)
PA(36:4)
PE(O−40:5)
LPC(O−16:1)
LPC(O−17:0)
TG(47:1)
PA(38:4)
Cer(d34:1)
LPA(16:0)
DG(44:12)
PC(O−40:6)
LPC(20:5)
SM(t30:1)
PS(40:6)
TG(49:1)
LPC(O−24:4)
PC(42:2)
SM(d39:3)
PC(O−41:4)
LPE(22:6)
DG(36:5)
PE(O−40:6)
TG(56:11)
PE(O−41:5)
TG(51:2)
DG(36:2)
DG(38:5)
DG(36:1)
PS(34:1)
TG(44:3)
DG(34:2)
TG(54:6)
PE(O−34:4)
PE(O−40:4)
LPC(18:2)
PC(44:10)
PC(O−36:4)
DG(36:4)
LPC(24:0)
PC(36:2)
TG(49:0)
TG(55:0)
SM(d40:2)
0.00
0.02
0.04
0.06
0.08
0.10
MeanDecreaseGini
